# Online Functional Metacognitive Intervention for Work-Performance Improvement in Adults with Attention Deficit Hyperactivity Disorder

**DOI:** 10.1192/j.eurpsy.2022.449

**Published:** 2022-09-01

**Authors:** N. Grinblat, S. Rosenblum

**Affiliations:** University of Haifa, Department Of Occupational Therapy Faculty Of Social Welfare & Health Sciences, Haifa, Israel

**Keywords:** Online Therapy, Work-Performance, Attention Deficit Hyperactivity Disorder

## Abstract

**Introduction:**

Adult attention deficit hyperactivity disorder (ADHD) is associated with reduced work performance. Online interventions increase accessibility of services to clients by removing barriers such as physical distance, which may prevent care.

**Objectives:**

This study aimed to assess the efficacy of an innovative functional metacognitive intervention for work-performance improvement of adults with ADHD.

**Methods:**

This study used a wait-list control group design, with a study and a comparison group (total 46 adults, mean age of 35.65 years). All participants had been diagnosed with ADHD, worked at least 3 months at the same place, and were willing to improve their work performance. Intervention sessions were provided mostly online and focused on the adults’ occupational goals in a workplace context. The intervention’s efficacy was evaluated with a focus on participants’ work performance (Canadian Occupational Performance Measure) executive functions (Behavior Rating Inventory of Executive Function-Adult), organisation in time (Time Organisation and Participation Scale), and quality of life (Adult ADHD Quality of Life Questionnaire).

**Results:**

Participants’ work performance, executive functions, organisation in time and quality of life significantly improved following the intervention. Their achievements were maintained through to the 3-month follow-up.

**Conclusions:**

The online metacognitive functional intervention for work-performance improvement of adults with ADHD was found to be efficient and suitable for clinical use among this population. Future studies with larger samples and additional objective measures are needed to further validate these findings.

**Disclosure:**

No significant relationships.

